# Association Between Dietary Patterns and Different Metabolic Phenotypes in Japanese Adults: WASEDA'S Health Study

**DOI:** 10.3389/fnut.2022.779967

**Published:** 2022-01-27

**Authors:** Kumpei Tanisawa, Tomoko Ito, Ryoko Kawakami, Chiyoko Usui, Takuji Kawamura, Katsuhiko Suzuki, Shizuo Sakamoto, Kaori Ishii, Isao Muraoka, Koichiro Oka, Mitsuru Higuchi

**Affiliations:** ^1^Faculty of Sport Sciences, Waseda University, Tokorozawa, Japan; ^2^Waseda Institute for Sport Sciences, Tokorozawa, Japan; ^3^Department of Food and Nutrition, Tokyo Kasei University, Tokyo, Japan; ^4^Research Center for Molecular Exercise Science, University of Physical Education, Budapest, Hungary; ^5^Faculty of Sport Science, Surugadai University, Saitama, Japan

**Keywords:** diet, metabolically healthy obese, metabolically unhealthy non-obese, healthy dietary pattern, alcohol dietary pattern, Japanese adults

## Abstract

Although many studies have reported that a posteriori dietary pattern is associated with metabolic health, there is little evidence of an association between dietary patterns and different metabolic phenotypes. The present study aimed to examine the association between major dietary patterns and different metabolic phenotypes (metabolically healthy non-obese [MHNO], metabolically unhealthy non-obese [MUNO], metabolically healthy obese [MHO], and metabolically unhealthy obese [MUO]) in middle-aged and elderly Japanese adults. This cross-sectional study enrolled 2,170 Japanese adults aged ≥40 years. The four different metabolic phenotypes were determined based on the presence of obesity, abdominal obesity, hypertension, hyperglycemia, and dyslipidemia. The major dietary patterns were determined using principal component analysis based on energy-adjusted food intake. Two dietary patterns were identified: the healthy dietary pattern, which was characterized by a high intake of vegetables, fruits, potatoes, soy products, mushrooms, seaweeds, and fish; and the alcohol dietary pattern, which was characterized by a high intake of alcoholic beverages, liver, chicken, and fish. The healthy dietary pattern was associated with the MHNO and MHO phenotypes (MUNO and MUO as reference groups, respectively), and the multivariate-adjusted odds ratios (ORs) (95% confidence intervals [CIs]) in the highest quartile of healthy dietary pattern score with the lowest quartile as the reference category were 2.10 (1.40–3.15) and 1.86 (1.06–3.25), respectively. Conversely, the alcohol dietary pattern was inversely associated with the MHNO and MHO phenotypes, while the multivariate-adjusted ORs (95% CIs) in the highest quartile of the alcohol dietary pattern score with the lowest quartile as the reference category were 0.63 (0.42–0.94) and 0.45 (0.26–0.76), respectively. There were no significant interactions between sex and healthy/alcohol dietary patterns in the prevalence of the MHNO and MHO phenotypes. In conclusion, the present study's findings suggest that major dietary patterns are associated with different metabolic phenotypes in middle-aged and elderly Japanese adults. These findings provide useful evidence for maintaining metabolic health through diet regardless of obesity status.

## Introduction

Obesity and abdominal obesity are associated with an increased risk of metabolic abnormalities such as hypertension, hyperglycemia, and dyslipidemia, which are associated with a higher incidence of cardiovascular diseases and mortality (1). Therefore, the prevention and improvement of obesity and abdominal obesity are among the most important factors in maintaining metabolic health. However, substantial heterogeneity in metabolic abnormalities exists among obese individuals, and an obese phenotype without metabolic abnormalities is called metabolically healthy obesity (MHO) (2). On the other hand, heterogeneity in metabolic abnormalities also exists among non-obese individuals. A non-obese phenotype with metabolic abnormalities is referred to as metabolically unhealthy normal weight (MUNW) (also referred to as metabolically unhealthy non-obese [MUNO]) (3). These concepts have attracted more attention recently because investigating them may increase our understanding of the pathophysiology of cardiometabolic diseases and aid in the establishment of effective treatments for them.

A meta-analysis of cohort studies demonstrated no significantly increased risk of all-cause mortality among MHO individuals vs. metabolically unhealthy non-obese (MHNO) individuals (4). On the other hand, a large-scale cohort study reported that normal-weight individuals with metabolic abnormalities were at an increased risk of cardiovascular diseases compared to those without metabolic abnormalities (5). Furthermore, several studies reported that the risk of cardiovascular disease mortality of individuals with MUNO is comparable to that of metabolically unhealthy obese (MUO) individuals (6, 7). These findings highlight the importance of maintaining metabolic health regardless of obesity status for the prevention of cardiovascular diseases.

Diet plays an important role in maintaining metabolic health. Although traditional nutritional epidemiology studies focused on the intake of single nutrients and foods, recent studies have performed dietary pattern analyses to capture participants' overall diet because people eat meals consisting of a variety of foods with complex combinations of nutrients that may be interactive or synergistic (8). Many studies have explored the associations between posteriori (data-driven) dietary patterns and metabolic health using statistical methods such as principal component analysis, factor analysis, and cluster analysis. A meta-analysis of the association between a posteriori dietary pattern and the prevalence of metabolic syndrome showed that a Western/unhealthy dietary pattern was positively associated with an increased prevalence of metabolic syndrome, but an inverse association between a prudent/healthy dietary pattern and metabolic syndrome was observed (9, 10). However, there is little evidence of an association between dietary patterns and different metabolic phenotypes, such as MHO and MUNO. Furthermore, few studies have investigated the association between dietary patterns and different metabolic phenotypes in East Asian populations, including those in Japan. Because the characteristics of posteriori dietary patters vary among populations with limited generalizability (11), it is important to investigate whether a posteriori dietary pattern is associated with different metabolic phenotypes in the Japanese population. Therefore, the present study aimed to examine the association between major dietary patterns and different metabolic phenotypes in middle-aged and elderly Japanese adults.

## Methods

### Participants

This cross-sectional study used baseline survey data from the Waseda Alumni's Sports, Exercise, Daily Activity, Sedentariness, and Health Study (WASEDA'S Health Study), which is a prospective cohort study among the alumni of Waseda University and their spouses aged ≥40 years (12–14). The WASEDA'S Health Study consists of four cohorts (cohorts A–D) with different measurement items, and the participants selected one of the four cohorts when registering for the study. The present study comprised a total of 2,544 individuals (men: *n* = 1,614; women: *n* = 930) who participated in the baseline survey of cohort C or D between March 2015 and March 2020. We excluded participants who met the following criteria: (1) a history of heart diseases (*n* = 196); (2) incomplete web-based questionnaires (*n* = 93); (3) incomplete dietary survey (*n* = 19); (4) extreme self-reported energy intake (<600 kcal/day or ≥4,000 kcal/day) (*n* = 10); (5) extreme self-reported physical activity (*n* = 26); (6) consumption of breakfast before blood sampling (*n* = 22); (7) lack of blood biochemical parameters (*n* = 7); and (8) non-Japanese ethnicity (*n* = 1). Based on the above criteria, 2,170 Japanese adults (men, *n* = 1,354; mean age, 55.5 [standard deviation, 10.1] years; women, *n* = 816, mean age, 51.5 [standard deviation 8.1] years) were included in the analysis. All participants provided written informed consent before enrolling in the study, which was approved by the Ethical Review Committee of Waseda University (reference numbers 2014-095, 2014-G002, 2018-320, and 2018-G001). The study was conducted in accordance with the principles of the Declaration of Helsinki.

### Health Examination

The participants visited the laboratory between 8:30 am and 10:30 am, and all measurements were conducted by trained investigators. Height was measured using a stadiometer (YHS-200D, YAGAMI Inc., Nagoya, Japan). Body weight was measured using an electronic scale (MC-980A, Tanita Corp., Tokyo, Japan) with the participants wearing light clothing and no shoes. Body mass index (BMI) was calculated as body weight (kg) divided by the body height in meters squared (m^2^). Waist circumference was measured to the nearest 0.1 cm at the umbilical region with an inelastic measuring tape at the end of normal expiration.

Venous blood samples were collected by venipuncture after at least 12 h of overnight fasting. Blood samples were collected into serum separation tubes or ethylenediaminetetraacetic acid–containing tubes and subsequently centrifuged at 3,000 rpm at 4°C for 10 min using a centrifuge (Model 5911, Kubota, Tokyo, Japan). Serum levels of aspartate aminotransferase (AST), alanine aminotransferase (ALT), γ-glutamyl transferase (γ-GTP), triglycerides, high-density lipoprotein (HDL) cholesterol, low-density lipoprotein (LDL) cholesterol, and fasting insulin as well as plasma levels of fasting glucose were determined using standard laboratory methods at BML, Inc. (Tokyo, Japan). Homeostasis model assessment of insulin resistance (HOMA-IR) was calculated from the fasting concentrations of plasma glucose and serum insulin as follows:


$$\begin{array}{l} \text{HOMA}-\text{IR}=\left(\text{fasting glucose}\left[\text{mg}/\text{dL}\right]\right)\\ \;\times \left(\text{fasting glucose}\left[\mu \text{U}/\text{mL}\right]\right)/405(15). \end{array}$$

Brachial systolic and diastolic blood pressures were measured using the oscillometric method (HEM-7122; OMRON, Inc, Kyoto, Japan) with the participants at rest in a seated position. Physical activity, marital status (yes or no), educational status (higher than college or not), household income (higher than 5,000,000 JPY or not), and smoking status (current, former, or never smoker) were assessed via a web-based questionnaire survey. Moderate to vigorous physical activity (MVPA) was assessed using the Global Physical Activity Questionnaire (16) to quantify the physical activity level, and the time spent in total MVPA (min/day) was calculated.

### Dietary Assessment

Dietary intake was assessed using a validated brief self-administered diet history questionnaire (BDHQ) in the preceding month, as described previously (12–14). The BDHQ is a 4-page questionnaire that takes ~15 min to complete. The dietary intake of 58 food and beverage items, energy, and selected nutrients was estimated using an *ad hoc* computer algorithm for the BDHQ, based on the Standard Tables of Food Composition in Japan (17). The validity of the dietary intake data (energy, nutrients, and foods) assessed by the BDHQ was confirmed using 16-day semi-weighted dietary records as the gold standard (18, 19). We carefully checked each completed BDHQ to avoid the effects of misreporting.

### Definition of Obesity and Abdominal Obesity

Obesity was defined as a BMI ≥25 kg/m^2^ for men and women according to the criteria established by the Japan Society for the Study of Obesity (20). Abdominal obesity in men was defined as a waist circumference ≥85 cm according to the criteria of abdominal obesity for the diagnosis of metabolic syndrome in Japan (21). Although a waist circumference ≥90 cm is used as a cut-off value for the diagnosis of metabolic syndrome in Japanese women (21), we defined abdominal obesity for women as a waist circumference ≥80 cm for the following reasons: (1) several studies suggested the optimal cut-off value to yield maximum sensitivity and specificity for predicting the presence of multiple metabolic syndrome components was approximately 80 cm for Japanese women (22–24); and (2) the cut-off value of waist circumference proposed by the International Diabetes Federation (IDF) is 80 cm for Asian women, including Japanese women (25). The participants were divided into non-obese (without obesity or abdominal obesity) and obese (with obesity and/or abdominal obesity) groups according to the criteria described above.

### Definition of Metabolic Phenotypes

A metabolically healthy phenotype was defined as the absence of any metabolic syndrome components as defined by IDF criteria (25): hypertension (systolic blood pressure ≥130 mmHg, diastolic blood pressure ≥85 mmHg or use of antihypertensive drugs), hyperglycemia (fasting glucose level ≥100 mg/dL or use of glucose-lowering drugs), dyslipidemia (low HDL cholesterol levels (<40 mg/dL for men and <50 mg/dL for women), or high triglyceride levels (≥150 mg/dL or use of triglyceride-lowering drugs). Thus, individuals in the non-obese group with no components were defined as having the MHNO phenotype, whereas those in the obese group with no components were defined as having the MHO phenotype. A metabolically unhealthy phenotype was defined as the presence of at least one of the metabolic syndrome components described above. Thus, individuals in the non-obese group with one or more components were defined as having the MUNO phenotype, whereas individuals in the obese group with one or more components were defined as having the MUO phenotype.

### Statistical Analysis

To identify major dietary patterns, we performed a principal component analysis based on energy-adjusted food intake using a density method of 52 food and beverage items as described previously (12–14). We retained the factors with eigenvalues >2.5 and discarded other factors based on the results of a screening test and the interpretability of the factors. The differences in the characteristics between different metabolic phenotypes (MHNO vs. MUNO and MHO vs. MUO) were assessed by Student's *t*-test (for continuous variables) and the χ^2^ test (for categorical variables). The differences in daily nutrient intake according to dietary pattern score quartile were assessed using linear regression analysis. To evaluate the associations between each dietary pattern and the prevalence of metabolically healthy phenotypes in the non-obese and obese groups, respectively, we performed a logistic regression analysis and calculated the age-adjusted and multivariate-adjusted odds ratios (ORs) and 95% confidence intervals (CIs) for the prevalence of metabolically healthy phenotypes according to the quartile of each dietary pattern score using the lowest quartile as the reference category. Since variables such as socioeconomic and lifestyle habits were potentially related to both dietary patterns and a metabolically healthy status, Model 1 was adjusted for age, sex, marital status, educational status, household income, smoking status, MVPA, and energy intake. Model 2 was additionally adjusted for waist circumference and HOMA-IR to evaluate whether the observed association was mediated by abdominal obesity and insulin resistance. We additionally performed a subgroup analysis by sex and added an interaction term (sex × dietary pattern [quartile]) to the model to test the significance of the interaction. We also performed a sensitivity analysis using different numbers of metabolic syndrome criteria for defining metabolic phenotypes because previous studies have shown that the estimated prevalence of MHO varies depending on the number of metabolic syndrome criteria used (26). In the sensitivity analysis, a metabolically healthy phenotype was defined as the presence of zero or one metabolic syndrome component. The level of statistical significance was set at P < 0.05. All statistical analyses were performed using SPSS Statistics (version 27.0; SPSS, Inc., Chicago, IL, USA).

## Results

### Dietary Patterns

We identified two dietary patterns using principal component analysis (Table 1). The first factor was named the healthy dietary pattern because it was characterized by a high intake of vegetables, fruits, potatoes, soy products, mushrooms, seaweeds, and fish. The second factor was named the alcohol dietary pattern because it was characterized by a high intake of alcoholic beverages, liver, chicken, and fish. The first and second dietary patterns explained 15.2% of the variance in food intake.

**Table 1:** Factor loading matrix for dietary patterns identified by the principal component analysis.

**Food groups**	**Healthy dietary**	**Alcohol dietary**
	**pattern**	**pattern**
Low fat Milk		
Milk/yogurt	0.15	−0.22
Chicken	0.19	0.26
Pork/beef		
Hum/sausage/bacon		
Liver		0.31
Squid/octopus/shrimps/shellfish	0.18	0.28
Small fish with bone	0.31	0.26
Canned tuna		
Dried fish/salted fish	0.23	0.17
Oily fish	0.29	0.22
Lean fish	0.31	0.20
Egg	0.27	
Tofu/atsuage^†^	0.45	
Natto^*^	0.25	
Potatoes	0.35	
Pickled green leaves vegetable	0.20	0.17
Pickled other vegetables	0.16	
Lettuces/cabbage (raw)	0.44	
Green leaves vegetable	0.70	
Cabbage/Chinese cabbage	0.66	
Carrots/pumpkin	0.71	
Japanese radish/turnip	0.64	
Other root vegetables	0.68	
Tomatoes	0.43	
Mushrooms	0.65	
Seaweeds	0.55	
Western–type confectioneries		−0.59
Japanese confectioneries		−0.42
Rice crackers/rice cake/okonomiyaki^‡^		−0.43
Ice cream		−0.25
Citrus fruit	0.27	−0.25
Persimmons/strawberries/kiwifruit	0.33	−0.22
Other fruit	0.39	−0.26
Mayonnaise/dressing		
Bread		−0.51
Japanese noodles		0.24
Buckwheat noodles		
Chinese noodle	−0.32	
Pasta	−0.18	
Green tea	0.16	
Black tea/oolong tea		
Coffee		
Cola drink/soft drink	−0.18	
100% fruit and vegetable juice		
Rice	−0.33	
Miso soup		
Sake		0.36
Beer	−0.28	0.43
Shochu	−0.22	0.43
Whisky	−0.15	0.28
Wine		0.33
Variance explained (%)	10.2	5.0

*A factor loading less than ± 0.15 is not shown*.

* *Fermented soybeans*.

† *Deep–fried tofu*.

‡ *Savory pancake with various ingredients (meat, fish, and vegetables)*.

### Participant Characteristics According to Different Metabolic Phenotypes by Sex

We compared the characteristics of the participants with different metabolic phenotypes (Table 2). In men, the prevalence of the MHNO, MUNO, MHO, and MUO phenotypes was 22.5, 31.5, 6.3, and 39.7%, respectively. MUNO individuals showed significantly higher age, BMI, waist circumference, systolic blood pressure, diastolic blood pressure, AST, ALT, γ-GTP, triglycerides, fasting glucose, fasting insulin, and HOMA-IR but significantly shorter height than MHNO individuals. These differences were also observed between the MHO and MUO individuals. Marital and smoking status differed significantly between MHNO and MUNO individuals, whereas educational status differed significantly between MHO and MUO individuals.

**Table 2:** Participants' characteristics according to metabolic phenotype in men vs. women.

	**Men**						**Women**					
	**MHNO**	**MUNO**	***P*–value**	**MHO**	**MUO**	***P*–value**	**MHNO**	**MUNO**	***P*–value**	**MHO**	**MUO**	***P*–value**
n	305	426		85	538		389	118		137	172	
Age (years)	51.8 (9.1)	57.3 (10.2)	< 0.001	52.6 (9.1)	56.7 (10.1)	0.001	49.7 (7.1)	53.6 (8.3)	< 0.001	51.2 (6.9)	54.6 (9.5)	0.001
Height (cm)	170.7 (5.0)	169.1 (5.8)	< 0.001	172.8 (6.3)	170.7 (5.8)	0.002	159.0 (5.3)	159.3 (5.2)	0.631	159.3 (5.1)	158.2 (5.8)	0.077
Body weight (kg)	62.6 (5.7)	63.0 (5.9)	0.342	73.9 (6.9)	75.7 (9.0)	0.077	49.6 (5.1)	51.3 (5.2)	0.002	59 (5.7)	61.8 (10.0)	0.003
BMI (kg/m^2^)	21.5 (1.6)	22.0 (1.6)	< 0.001	24.8 (2.1)	26.0 (2.6)	< 0.001	19.6 (1.7)	20.2 (1.7)	0.001	23.2 (1.9)	24.7 (3.7)	< 0.001
Waist circumference (cm)	77.2 (4.7)	79.1 (4.3)	< 0.001	88.7 (4.1)	91.7 (6.1)	< 0.001	71.2 (4.8)	73.5 (4.2)	< 0.001	85.3 (4.6)	87 (7.1)	0.012
Systolic blood pressure (mmHg)	113.9 (8.7)	136.0 (17.1)	< 0.001	116.0 (7.5)	139.0 (18.4)	< 0.001	107.1 (10.9)	132.7 (19.7)	< 0.001	111.7 (10.4)	133.2 (20.0)	< 0.001
Diastolic blood pressure (mmHg)	71.0 (6.6)	84.4 (11.4)	< 0.001	73.6 (6.9)	87.0 (11.8)	< 0.001	67.2 (7.8)	83.0 (12.8)	< 0.001	70.7 (7.9)	83.1 (11.0)	< 0.001
AST (U/L)	22.8 (5.3)	25.4 (10.7)	< 0.001	23.0 (5.9)	26.5 (9.9)	0.002	21.6 (6.5)	22.5 (6.7)	0.188	20.6 (6.0)	23.3 (7.7)	0.001
ALT (U/L)	21.0 (7.9)	23.4 (11.1)	0.001	24.3 (10.5)	30.2 (17.9)	0.003	17.3 (9.4)	17.8 (6.8)	0.625	18.2 (11.3)	24.4 (14.6)	< 0.001
γ-GTP (U/L)	30.8 (21.7)	45.9 (54.8)	< 0.001	42.0 (36.5)	56.5 (56.3)	0.022	21.7 (16.4)	25.3 (20.8)	0.049	24.2 (19.7)	34.3 (31.0)	0.001
Triglycerides (mg/dL)	73.2 (28.3)	111.3 (102.1)	< 0.001	92.2 (29.0)	145.3 (109.7)	< 0.001	61.9 (23.4)	83.6 (59.4)	< 0.001	73.9 (22.8)	109.3 (63.1)	< 0.001
HDL cholesterol (mg/dL)	68.4 (14.7)	66.2 (17.1)	0.063	59.1 (11.7)	57.0 (13.4)	0.171	80.4 (15.2)	77.0 (17.2)	0.042	71.5 (13.0)	67.5 (16.3)	0.020
LDL cholesterol (mg/dL)	121.5 (28.1)	125 (29.9)	0.103	130.9 (25.8)	129.5 (29.9)	0.672	117.0 (31.5)	123.4 (32.8)	0.055	133.6 (27.4)	133.0 (34.6)	0.882
Fasting glucose (mg/dL)	88.9 (5.8)	98.7 (15)	< 0.001	91.0 (4.9)	101.1 (17.6)	< 0.001	86.3 (6.2)	92.1 (8.9)	< 0.001	88.0 (5.9)	96.8 (14.3)	< 0.001
Fasting insulin (μU/mL)	3.7 (1.7)	5.0 (2.9)	< 0.001	6.0 (2.9)	7.7 (4.4)	< 0.001	3.7 (1.7)	4.6 (2.4)	< 0.001	4.9 (2.0)	7.6 (4.7)	< 0.001
HOMA–IR	0.8 (0.4)	1.2 (0.8)	< 0.001	1.3 (0.6)	2.0 (1.3)	< 0.001	0.8 (0.4)	1.1 (0.6)	< 0.001	1.1 (0.5)	1.8 (1.3)	< 0.001
MVPA (min/day)	53.7 (54.9)	58.5 (48.6)	0.217	45.8 (36.7)	49.5 (52.6)	0.528	46.8 (56.8)	51.2 (45.5)	0.449	43.7 (55.2)	40.7 (41.7)	0.581
Marital status (% married)	83.3	91.1	0.001	91.8	87.0	0.214	76.3	80.5	0.345	85.4	80.2	0.235
Educational status (% with college diploma)	99.0	98.8	0.808	95.3	98.9	0.014	78.1	80.5	0.583	75.9	80.8	0.296
Household income (% ≥5,000,000 JPY)	82.0	79.6	0.421	81.2	77.5	0.448	79.4	73.7	0.189	80.3	72.1	0.095
Smoking status			0.001			0.310			0.614			0.320
Current smoker (%)	6.6	7.3		8.2	13.8		2.3	2.5		2.2	3.5	
Former smoker (%)	33.4	48.6		42.4	42.9		13.6	10.2		13.1	18.6	
Never–smoker (%)	60.0	44.1		49.4	43.3		84.1	87.3		84.7	77.9	
Hypertension (%)	46.1			72.9			18.5			40.1		
Hyperglycemia (%)	23.5			37.2			4.7			18.8		
Dyslipidemia (%)	13.5			35.2			3.7			16.2		

*ALT, alanine aminotransferase; AST, aspartate aminotransferase; BMI, body mass index; γ-GTP, γ-glutamyl transpeptidase; HDL cholesterol, high-density lipoprotein cholesterol; HOMA-IR, homeostasis model assessment of insulin resistance; LDL cholesterol, low-density lipoprotein cholesterol; MVPA, moderate and vigorous intensity physical activity. Data are presented as mean (standard deviation) for continuous variables and percentage for categorical variables*.

*P values were calculated using Student's t-test for continuous variables and the χ^2^ square test for categorical variables*.

*The level of statistical significance was set at P < 0.05*.

In women, the prevalence of MHNO, MUNO, MHO, and MUO phenotypes was 47.7, 14.5, 16.8, and 21.1%, respectively. Compared with MHNO individuals, MUNO individuals showed significantly higher age, body weight, BMI, waist circumference, systolic blood pressure, diastolic blood pressure, γ-GTP, triglycerides, fasting glucose, fasting insulin, and HOMA-IR but significantly lower HDL cholesterol. These differences were also observed between MHO and MUO individuals. AST and ALT levels were significantly higher in MUO individuals than in MHO individuals.

### Daily Nutrient Intake According to Dietary Pattern Score Quartile

The daily nutrient intake according to the dietary pattern score quartiles in men is shown in Table 3. A higher healthy dietary pattern score was associated with a higher intake of all nutrients except energy intake and lower energy percentages from carbohydrates and alcohol. A higher alcohol dietary pattern score was associated with higher energy intake, higher energy percentage from protein, alcohol, and n-3 polyunsaturated fatty acids, and higher energy-adjusted intake of sodium, magnesium, iron, vitamin A, vitamin D, and folate but lower energy percentages from fat, carbohydrate, and saturated fatty acids and energy-adjusted intake of calcium, vitamin C, and dietary fiber.

**Table 3:** Daily nutrient intake according to dietary pattern score quartile in men vs. women.

		**Healthy dietary pattern**			**Alcohol dietary pattern**		
		**Q1**	**Q4**	***P*–value**	**Q1**	**Q4**	***P*–value**
Men						
	Energy intake (kcal)	2070 (549)	2066 (536)	0.684	1993 (503)	2086 (533)	0.035
	Protein (% energy)	13.2 (1.9)	18.8 (3.3)	<0.001	14.6 (1.8)	15.9 (3.7)	< 0.001
	Fat (% energy)	24.1 (5.1)	30.7 (6.6)	<0.001	28.9 (4.9)	26.0 (6.1)	< 0.001
	Carbohydrate (% energy)	50.4 (9.3)	45.9 (9.7)	<0.001	54.2 (5.9)	42.1 (8.6)	< 0.001
	Alcohol (% energy)	10.7 (10)	4.3 (5.3)	<0.001	1.6 (2.5)	14.9 (9.5)	< 0.001
	Saturated fatty acids (% energy)	6.4 (1.8)	8.0 (2.0)	<0.001	8.3 (1.9)	6.5 (1.8)	< 0.001
	n-3 polyunsaturated fatty acids (% energy)	1.1 (0.3)	1.7 (0.5)	<0.001	1.2 (0.3)	1.5 (0.4)	< 0.001
	n-6 polyunsaturated fatty acids (% energy)	4.8 (1.0)	5.9 (1.5)	<0.001	5.4 (1.0)	5.2 (1.3)	0.099
	Sodium (mg/1,000 kcal)	2154 (408)	2534 (534)	<0.001	2205 (380)	2396 (498)	< 0.001
	Potassium (mg/1,000 kcal)	1109 (192)	2021 (335)	<0.001	1428 (335)	1439 (403)	0.956
	Calcium (mg/1,000 kcal)	223 (69)	420 (104)	<0.001	310 (88)	289 (112)	0.005
	Magnesium (mg/1,000 kcal)	119 (18)	186 (28)	<0.001	136 (25)	149 (35)	< 0.001
	Iron (mg/1,000 kcal)	3.5 (0.6)	5.9 (1.0)	<0.001	4.2 (0.9)	4.6 (1.2)	< 0.001
	Vitamin A (μg RAE/1,000 kcal)^*^	348 (210)	618 (248)	<0.001	390 (159)	498 (286)	< 0.001
	Vitamin D (μg/1,000 kcal)	5.3 (2.5)	10.7 (5.9)	<0.001	5.7 (2.6)	8.8 (5.2)	< 0.001
	Folate (μg/1,000 kcal)	142 (33)	284 (70)	<0.001	185 (56)	198 (69)	0.012
	Vitamin C (mg/1,000 kcal)	42.9 (16.7)	99.3 (31.6)	<0.001	68.6 (30.8)	58.1 (26.4)	< 0.001
	Dietary fiber (g/1,000 kcal)	4.9 (1.0)	9.5 (2.2)	<0.001	6.7 (1.8)	6.3 (2.1)	< 0.001
Women						
	Energy intake (kcal)	1606 (464)	1769 (465)	<0.001	1702 (471)	1725 (481)	0.640
	Protein (% energy)	13.0 (1.7)	18.7 (3.1)	<0.001	15.3 (1.9)	18.6 (5.3)	< 0.001
	Fat (% energy)	25.5 (5.5)	31.3 (5.7)	<0.001	29.6 (4.3)	29.2 (8.7)	0.974
	Carbohydrate (% energy)	52.9 (9.4)	47.4 (8.4)	<0.001	53.6 (5.4)	39.7 (9.5)	< 0.001
	Alcohol (% energy)	7.1 (10.3)	2.4 (4.5)	<0.001	1.0 (1.9)	11.6 (10.7)	< 0.001
	Saturated fatty acids (% energy)	7.1 (2.0)	8.4 (1.7)	<0.001	8.6 (1.6)	7.5 (2.4)	< 0.001
	n−3 polyunsaturated fatty acids (% energy)	1.1 (0.3)	1.6 (0.4)	<0.001	1.3 (0.3)	1.7 (0.5)	< 0.001
	n−6 polyunsaturated fatty acids (% energy)	4.8 (1.1)	5.9 (1.3)	<0.001	5.4 (1.0)	5.5 (1.9)	0.034
	Sodium (mg/1,000 kcal)	2031 (381)	2401 (497)	< 0.001	2129 (358)	2547 (646)	< 0.001
	Potassium (mg/1,000 kcal)	1091 (172)	2032 (348)	<0.001	1561 (370)	1773 (561)	<0.001
	Calcium (mg/1,000 kcal)	229 (74)	424 (100)	<0.001	337 (97)	362 (149)	0.006
	Magnesium (mg/1,000 kcal)	115 (17)	185 (30)	<0.001	145 (28)	174 (46)	< 0.001
	Iron (mg/1,000 kcal)	3.4 (0.6)	5.9 (1.1)	<0.001	4.5 (0.9)	5.4 (1.7)	< 0.001
	Vitamin A (μg RAE/1,000 kcal)^*^	311 (195)	622 (259)	<0.001	427 (159)	650 (465)	< 0.001
	Vitamin D (μg/1,000 kcal)	4.6 (3.1)	10.1 (5.3)	<0.001	6.1 (3.2)	11.3 (7.2)	< 0.001
	Folate (μg/1,000 kcal)	135 (33)	288 (75)	<0.001	206 (64)	245 (100)	< 0.001
	Vitamin C (mg/1,000 kcal)	39.5 (14)	101.3 (30.4)	<0.001	76.6 (31.4)	71.0 (32.9)	0.327
	Dietary fiber (g/1,000 kcal)	5.0 (1.0)	9.7 (2.1)	<0.001	7.5 (1.9)	7.6 (3.0)	0.113

*RAE, retinol activity equivalent*.

*Data are presented as mean (standard deviation)*.

*P values were calculated using linear regression analysis*.

*The level of statistical significance was set at P < 0.05*.

* *1 μg RAE = retinol (μg) + β-carotene (μg) × 1/12 + α-carotene (μg) × 1/24 + β-cryptoxanthin (μg) × 1/24 + other provitamin A carotenoids (μg) × 1/24*.

The daily nutrient intake of women is shown in Table 3. The associations of nutrient intake with a healthy dietary pattern score were the same between men and women except for energy intake, which was positively associated with the healthy dietary pattern score only in women. The associations of most nutrient intakes with the alcohol dietary pattern score were similar between men and women. However, the alcohol dietary pattern score was positively associated with the energy percentage of n-6 polyunsaturated fatty acids and energy-adjusted intakes of potassium and calcium in women, but there were no associations or inverse associations of these variables with the alcohol dietary pattern in men.

### Association Between Dietary Patterns and Prevalence of the MHNO Phenotype in the Non-obese Group

Table 4 shows the ORs and 95% CIs for the prevalence of the MHNO phenotype according to dietary pattern score quartiles in the non-obese group. A logistic regression model adjusted for potential confounders (Model 1) showed that a healthy dietary pattern was positively associated with the prevalence of the MHNO phenotype in men and women combined (P for trend < 0.001), and this association remained significant after the further adjustment for waist circumference and HOMA-IR in Model 2 (P for trend < 0.001).

**Table 4:** Odds ratios and 95% confidence intervals for the prevalence of the MHNO phenotype according to dietary pattern score quartile in the non-obese group.

		**Healthy dietary pattern**					**Alcohol dietary pattern**			
		**n**	**Cases**	**Model 1**	**Model 2**		**n**	**Cases**	**Model 1**	**Model 2**
				**OR (95% CI)**	**OR (95% CI)**				**OR (95% CI)**	**OR (95% CI)**
Men and women combined							
	Q1	295	143	1.00 (Reference)	1.00 (Reference)		336	221	1.00 (Reference)	1.00 (Reference)
	Q2	293	149	1.25 (0.87–1.79)	1.40 (0.96–2.04)		331	193	0.98 (0.69–1.39)	0.99 (0.68–1.44)
	Q3	305	171	1.46 (1.01–2.10)	1.50 (1.02–2.21)		315	167	0.90 (0.63–1.30)	0.96 (0.65–1.40)
	Q4	345	231	2.17 (1.48–3.19)	2.10 (1.40–3.15)		256	113	0.68 (0.46–1.00)	0.63 (0.42–0.94)
	P for trend			<0.001	<0.001				0.051	0.033
Men									
	Q1	221	86	1.00 (Reference)	1.00 (Reference)		123	55	1.00 (Reference)	1.00 (Reference)
	Q2	205	87	1.37 (0.91–2.07)	1.52 (0.98–2.36)		193	91	1.16 (0.71–1.88)	1.19 (0.71–1.99)
	Q3	172	71	1.50 (0.96–2.33)	1.55 (0.97–2.48)		215	87	0.88 (0.55–1.42)	0.94 (0.56–1.57)
	Q4	133	61	1.97 (1.21–3.21)	1.76 (1.05–2.96)		200	72	0.72 (0.44–1.17)	0.64 (0.38–1.09)
	P for trend			0.007	0.028				0.061	0.028
Women									
	Q1	74	57	1.00 (Reference)	1.00 (Reference)		213	166	1.00 (Reference)	1.00 (Reference)
	Q2	88	62	0.95 (0.46–2.00)	1.12 (0.51–2.45)		138	102	0.78 (0.46–1.30)	0.76 (0.44–1.31)
	Q3	133	100	1.33 (0.66–2.71)	1.48 (0.71–3.08)		100	80	1.12 (0.61–2.07)	1.06 (0.56–2.01)
	Q4	212	170	2.40 (1.17–4.90)	2.66 (1.26–5.61)		56	41	0.67 (0.33–1.36)	0.66 (0.31–1.39)
	P for trend			0.002	0.003				0.540	0.485

*HOMA–IR, homeostasis model assessment of insulin resistance; MVPA, moderate and vigorous intensity physical activity*.

*Model 1 was adjusted for age, marital status, educational status, household income, smoking status, MVPA, and energy intake*.

*Model 2 was additionally adjusted for waist circumference and HOMA–IR*.

*P values were calculated using logistic regression analysis*.

*The level of statistical significance was set at P < 0.05*.

The fully adjusted ORs (95% CIs) of the prevalence of the MHNO phenotype for the lowest to the highest quartiles of the healthy dietary pattern score were 1.00 (reference), 1.40 (0.96–2.04), 1.50 (1.02–2.21), and 2.10 (1.40–3.15), respectively. Furthermore, the alcohol dietary pattern was inversely associated with the prevalence of the MHNO phenotype when the model was adjusted for waist circumference and HOMA-IR in Model 2 (P for trend = 0.033). The fully adjusted ORs (95% CIs) of the prevalence of the MHNO phenotype for the lowest to the highest quartiles of the alcohol dietary pattern score were 1.00 (reference), 0.99 (0.68–1.44), 0.96 (0.65–1.40), and 0.63 (0.42–0.94), respectively.

Next we performed a subgroup analysis according to sex (Table 4). A logistic regression analysis showed no significant interaction between sex and the healthy dietary pattern for the prevalence of the MHNO phenotype (P for interactio*n* = 0.514), and a healthy dietary pattern was positively associated with the prevalence of the MHNO phenotype in both men and women. There was no significant interaction between sex and the alcohol dietary pattern for the prevalence of the MHNO phenotype (P for interactio*n* = 0.325); however, a significant inverse association between the alcohol dietary pattern and prevalence of the MHNO phenotype was observed in men.

In a sensitivity analysis, the healthy dietary pattern score was not significantly associated with the prevalence of the MHNO phenotype in both sexes (Model 1: P for trend = 0.225; Model 2: P for trend = 0.406). The fully adjusted ORs (95% CIs) of the prevalence of the MHNO phenotype from the lowest to the highest quartiles of the healthy dietary pattern score were 1.00 (reference), 1.67 (1.00–2.79), 1.65 (0.97–2.8), and 1.23 (0.70–2.15), respectively. On the other hand, the alcohol dietary pattern score was inversely associated with the prevalence of the MHNO phenotype in both sexes (Model 1: P for trend = 0.047; Model 2: P for trend = 0.012). The fully adjusted ORs (95% CIs) of the prevalence of the MHNO phenotype from the lowest to the highest quartiles of the alcohol dietary pattern score were 1.00 (reference), 1.00 (0.56–1.79), 1.03 (0.58–1.84), and 0.50 (0.28–0.88), respectively.

### Association Between Dietary Patterns and Prevalence of the MHO Phenotype in the Obese Group

Table 5 shows the ORs and 95% CIs for the prevalence of the MHO phenotype according to dietary pattern score quartile in the obese group. A healthy dietary pattern was positively associated with the prevalence of the MHO phenotype in both men and women (Model 1: P for trend = 0.006; Model 2: P for trend = 0.014). The fully adjusted ORs (95% CIs) of the prevalence of the MHO phenotype for the lowest to the highest quartiles of the healthy dietary pattern score were 1.00 (reference), 1.04 (0.62–1.77), 1.45 (0.86–2.44), and 1.86 (1.06–3.25), respectively. Furthermore, the alcohol dietary pattern was inversely and significantly associated with the prevalence of the MHO phenotype (Model 1: P for trend = 0.009; Model 2: P for trend = 0.009). The fully adjusted ORs (95% CIs) of the prevalence of the MHO phenotype for the lowest to the highest quartiles of the alcohol dietary pattern score were 1.00 (reference), 0.69 (0.42–1.14), 0.84 (0.51–1.38), and 0.45 (0.26–0.76), respectively.

**Table 5:** Odds ratios and 95% confidence intervals for the prevalence of the MHO phenotype according to dietary pattern score quartile in the obese group.

		**Healthy dietary pattern**				**Alcohol dietary pattern**			
		**n**	**Cases**	**Model 1**	**Model 2**	**n**	**Cases**	**Model 1**	**Model 2**
				**OR (95% CI)**	**OR (95% CI)**			**OR (95% CI)**	**OR (95% CI)**
Men and women combined									
	Q1	247	41	1.00 (Reference)	1.00 (Reference)	207	73	1.00 (Reference)	1.00 (Reference)
	Q2	249	46	0.97 (0.59–1.60)	1.04 (0.62–1.77)	211	56	0.68 (0.43–1.08)	0.69 (0.42–1.14)
	Q3	238	67	1.52 (0.93–2.50)	1.45 (0.86–2.44)	226	54	0.82 (0.52–1.30)	0.84 (0.51–1.38)
	Q4	198	68	1.87 (1.10–3.18)	1.86 (1.06–3.25)	288	39	0.47 (0.29–0.77)	0.45 (0.26–0.76)
	P for trend			0.006	0.014			0.009	0.009
Men									
	Q1	218	30	1.00 (Reference)	1.00 (Reference)	92	18	1.00 (Reference)	1.00 (Reference)
	Q2	180	18	0.80 (0.42–1.52)	0.75 (0.38–1.46)	121	12	0.41 (0.18–0.92)	0.38 (0.16–0.89)
	Q3	143	20	1.34 (0.71–2.55)	1.20 (0.61–2.34)	166	29	0.77 (0.39–1.51)	0.70 (0.34–1.43)
	Q4	82	17	2.48 (1.21–5.12)	2.15 (1.01–4.58)	244	26	0.44 (0.22–0.87)	0.44 (0.21–0.90)
	P for trend			0.018	0.057			0.091	0.129
Women									
	Q1	29	11	1.00 (Reference)	1.00 (Reference)	115	55	1.00 (Reference)	1.00 (Reference)	
	Q2	69	28	1.33 (0.53–3.35)	2.03 (0.73–5.66)	90	44	0.90 (0.50–1.60)	1.03 (0.55–1.96)
	Q3	95	47	1.91 (0.78–4.64)	2.29 (0.87–6.03)	60	25	0.77 (0.40–1.48)	0.88 (0.42–1.80)
	Q4	116	51	1.97 (0.81–4.81)	2.49 (0.94–6.59)	44	13	0.43 (0.19–0.95)	0.38 (0.16–0.91)
	P for trend			0.095	0.113			0.048	0.059

*HOMA–IR, homeostasis model assessment of insulin resistance; MVPA, moderate and vigorous intensity physical activity*.

*Model 1 was adjusted for age, marital status, educational status, household income, smoking status, MVPA, energy intake*.

*Model 2 was additionally adjusted for waist circumference and HOMA–IR*.

*P values were calculated using logistic regression analysis*.

*The level of statistical significance was set at P < 0.05*.

There was no significant interaction between sex and the healthy dietary pattern for the prevalence of the MHO phenotype (Table 5; P for interactio*n* = 0.877), and the healthy dietary pattern score was positively associated with the prevalence of the MHO phenotype in both men and women, although these associations were not statistically significant in any model. No significant interaction between sex and the alcohol dietary pattern for the prevalence of the MHO phenotype was observed (P for interactio*n* = 0.921), while the alcohol dietary pattern was inversely associated with the prevalence of the MHO phenotype in both men and women, although these associations were not statistically significant in any model in men.

In a sensitivity analysis, the healthy dietary pattern score was not significantly associated with the prevalence of the MHO phenotype in both sexes (Model 1: P for trend = 0.055; Model 2: P for trend = 0.190). The fully adjusted ORs (95% CIs) of the prevalence of the MHO phenotype for the lowest to the highest quartiles of the healthy dietary pattern score were 1.00 (reference), 1.66 (1.06–2.59), 1.05 (0.66–1.67), and 1.73 (1.01–2.97), respectively. On the other hand, the alcohol dietary pattern score was inversely associated with the prevalence of the MHO phenotype in both sexes (Model 1: P for trend < 0.001; Model 2: P for trend < 0.001). The fully adjusted ORs (95% CIs) of the prevalence of the MHO phenotype for the lowest to the highest quartiles of the alcohol dietary pattern score were 1.00 (reference), 0.74 (0.43–1.28), 0.59 (0.34–1.00), and 0.31 (0.18–0.51), respectively.

## Discussion

To our knowledge, this is the first study to investigate the association between dietary patterns and different metabolic phenotypes in Japanese adults. We demonstrated that a healthy dietary pattern was positively associated with the prevalence of metabolically healthy phenotypes regardless of obesity status. We also reported that the alcohol dietary pattern was inversely associated with the prevalence of metabolically healthy phenotypes regardless of obesity status. These results suggest that major dietary patterns are associated with different metabolic phenotypes in middle-aged and elderly Japanese adults.

The present study identified two dietary patterns using principal component analysis, naming the healthy dietary pattern as the first factor (Table 1). Many studies have identified healthy (prudent) dietary patterns, and the majority reported a favorable association between healthy dietary patterns and metabolic health. A meta-analysis of observational studies demonstrated that a healthy dietary pattern is inversely associated with the prevalence of metabolic syndrome (9, 10). Although a limited number of studies have examined the association between dietary patterns and different metabolic phenotypes, such as MUNO and MHO, several studies have reported that a healthy dietary pattern is associated with them. In a cross-sectional study, Slagter et al. demonstrated that the “fruit, vegetables, and fish” dietary pattern, characterized by a high intake of vegetables, fruit, tea, fatty fish, lean fish, and fermented milk products, was positively associated with the MHO phenotype in the Dutch population (27). Suliga et al. reported that the prudent dietary pattern, characterized by a high intake of fruit, vegetables, cottage cheese, yogurt, low-fat milk, and whole grains, was inversely associated with metabolically obese normal-weight phenotype (synonymous with MUNO) in a Polish-Norwegian study (28). These results agree with those of the present study (Tables 4, 5). Conversely, several studies did not detect a significant association between healthy dietary patterns and metabolic phenotypes. Mirzababaei et al. showed that a healthy dietary pattern, characterized by a high intake of vegetables, fruits, legumes, nuts, starchy vegetables, low-fat dairy, meat, and olives, was not associated with MHO in the Iranian population (29). Nikniaz et al. showed that a healthy dietary pattern, characterized by a high intake of dairy products, fruits, fruit juices, vegetables, legumes, coffee, and tea, was not associated with MHO in the Iranian population (30). Pereira et al. reported that a healthy dietary pattern, characterized by a high intake of whole grains, skimmed milk, vegetables and fruits, and chicken and fish, was not associated with metabolically obese normal-weight phenotypes in Brazilian adults (31). The inconsistent results among these studies may be partly due to differences in the characteristics of dietary patterns identified in each study. Because the characteristics of a posteriori dietary pattern vary among populations, their generalizability is limited (11). In fact, the characteristics of a dietary pattern named “healthy” or “prudent” differ only slightly among studies as shown above. The healthy dietary pattern identified in the present study was characterized by a high intake of vegetables, fruits, potatoes, soy products, mushrooms, seaweeds, and fish (Table 1), which are common features among the healthy dietary patterns identified in the Japanese population (11). On the other hand, the healthy dietary patterns identified in other ethnic populations are often characterized by a high intake of poultry, fish, low-fat dairy, legumes, and whole grains in addition to vegetables and fruits (9, 32, 33). Although it is unknown which food items and nutrients contributed to the inconsistent results among these studies, the results of the present study are reasonable because a considerable number of studies have shown a favorable association between a healthy dietary pattern and cardiometabolic health outcomes in Japanese populations (13, 34–37), and this dietary pattern is relatively reproducible in different populations within a country (11).

One plausible explanation for the association between healthy dietary patterns and metabolically healthy phenotypes is the high intake of nutrients that benefit metabolic health. For example, a healthy dietary pattern was strongly associated with a high potassium intake in the present study (Table 3), which was mainly attributed to the high intake of vegetables and fruits. A high potassium intake has been shown to reduce blood pressure in hypertensive individuals (38, 39). Because the prevalence of hypertension in the present study was higher than that of hyperglycemia and dyslipidemia (Table 2), a high potassium intake might have contributed greatly to metabolically healthy phenotypes. A healthy dietary pattern was also associated with a high intake of dietary fiber and n-3 polyunsaturated fatty acids (Table 3), of which the health benefits are widely recognized. A meta-analysis of randomized controlled trials reported that a high intake of dietary fiber reduces systolic blood pressure, triglycerides, and fasting glucose (40). A meta-analysis of intervention studies also reported that fish oil or n-3 polyunsaturated fatty acid intake reduces blood triglyceride levels (41, 42). Although these nutrients may not significantly affect metabolic health, their additive effect may have led to the association between healthy dietary patterns and metabolically healthy phenotypes in the present study. Interestingly, the characteristics of the healthy dietary pattern in the present study were different from those of a priori healthy dietary patterns, such as the Dietary Approaches to Stop Hypertension (DASH) (43) and Mediterranean dietary patterns (44). Both DASH and Mediterranean dietary patterns are characterized by a low intake of saturated fatty acids, which could reduce blood pressure and blood cholesterol levels. However, a higher score of the healthy dietary pattern in the present study was associated with a higher intake of saturated fatty acids (Table 3) despite a better metabolic profile. A plausible explanation for this discrepancy is that the amount of saturated fatty acids intake in our study population was relatively lower than that in the general Western population. The mean percentage energy from saturated fatty acids in the highest quartile of the healthy dietary pattern score was 8.0% in men and 8.4% in women. These values are much lower than those reported in Western populations (45), and may be insufficient to cause adverse metabolic effects. Furthermore, the healthy dietary pattern in the present study was characterized by high sodium intake (Table 3), unlike the DASH dietary pattern, which is characterized by low sodium intake. Because high sodium intake is recognized as an important risk factor for hypertension, the positive association between the healthy dietary pattern score and sodium intake seems unreasonable. However, the healthy dietary pattern score was also associated with potassium intake (Table 3), and the strength of the association was stronger than that with association with sodium intake. Therefore, high potassium intake might negate the adverse effects of high sodium intake on blood pressure.

The present study also demonstrated that the alcohol dietary pattern was inversely associated with the MHNO and MHO phenotypes. Although few studies have investigated the association between alcohol dietary patterns and different metabolic phenotypes, several studies reported that this dietary pattern is associated with metabolic abnormalities (28, 46). The unfavorable association between alcohol dietary patterns and metabolic health can be explained by the high alcohol intake. A meta-analysis of randomized controlled trials showed that reducing alcohol intake lowers blood pressure in a dose-dependent manner (47). Given the fact that the prevalence of hypertension was higher than that of hyperglycemia and dyslipidemia in the present study (Table 2), it is plausible that the increase in blood pressure due to a heavy alcohol intake contributed to the association of the alcohol dietary pattern with the metabolically unhealthy phenotypes. In terms of dyslipidemia, excessive alcohol intake has been suggested to be associated with high circulating triglyceride levels (48), although alcohol intake is also known to have a beneficial effect on circulating HDL cholesterol levels (49). Overall, the participants in the present study had high HDL cholesterol levels, and the prevalence of a low HDL cholesterol level (3.8%) was lower than that of a high triglyceride level (16.2%). Therefore, the adverse effect of alcohol intake on triglyceride level may be superior to the alcohol-induced increase in HDL cholesterol, thereby contributing to the high prevalence of the MUNO and MUO phenotypes in the present study.

Although many studies on metabolically healthy and unhealthy phenotypes have been conducted, there is no consensus on the definitions of these phenotypes, and each study used different criteria (26). Therefore, it is difficult to interpret the findings of the present study and compare them with those of previous studies. Moreover, the definition of metabolically healthy and unhealthy phenotypes in previous studies on dietary patterns and these phenotypes were all different from each other (27–31), which might contribute to inconsistent findings from these studies. Smith et al. reviewed the definition in the available literature and showed that the estimated prevalence of MHO varied from 7 to 50% among people with obesity in North America and Europe (26). This large variability was mainly due to the number of metabolic syndrome criteria. Therefore, we performed a sensitivity analysis of different numbers of metabolic syndrome criteria to confirm the robustness of our findings. The alcohol dietary pattern score was inversely associated with a metabolically healthy phenotype regardless of obesity status when the metabolically healthy phenotype was defined as the presence of zero or one metabolic syndrome component. On the other hand, the healthy dietary pattern score was not significantly associated with metabolically unhealthy phenotypes in individuals without and with obesity in the sensitivity analysis. This suggests that the healthy dietary pattern identified in the present study was not associated with metabolic abnormalities alone.

In the present study, the association between healthy and alcohol dietary patterns with the metabolically healthy/unhealthy phenotypes remained significant after the adjustment for waist circumference and HOMA-IR in the non-obese and obese groups. These results suggest that these dietary patterns are associated with metabolic phenotypes independent of abdominal obesity and insulin resistance. This finding is particularly important for maintaining metabolic health in non-obese people with normal waist circumferences. Abdominal obesity and insulin resistance play central roles in the development of metabolic abnormalities (50). Therefore, reducing abdominal obesity is considered the most effective way to improve metabolic health. For example, in the current screening and education systems for metabolic syndrome in Japan, obese or abdominal obese individuals with one or more metabolic risk factors can receive health educational support from insurers to improve their obesity and abdominal obesity (51). However, non-obese individuals with a normal waist circumference are ineligible for health education support, even if they have multiple metabolic risk factors, because an effective intervention to improve metabolic abnormalities in non-obese individuals has not been established. Given the fact that metabolic abnormalities increase the risk of cardiovascular diseases and mortality regardless of obesity status (5–7), it is important to identify the factors associated with metabolic abnormalities in non-obese individuals. Therefore, the findings from the present study are important and will contribute to the maintenance of metabolic health in non-obese individuals.

The present study has several limitations. First, its cross-sectional design does not allow the inference of causality. Prospective cohort and interventional studies are required to elucidate the causal relationship between dietary patterns and the different metabolic phenotypes. Second, our study participants were alumni of the same university and their spouses in Japan, which might have introduced selection bias. Further investigations of representative populations are necessary to generalize our findings to the entire Japanese population and other populations. Third, we did not consider menopausal status in women due to missing data. Estrogen has blood pressure- and lipid-lowering effects (52), and postmenopausal women have higher blood pressure and triglyceride levels than premenopausal women (53). Therefore, menopausal status should be included as a covariate in future analyses.

In conclusion, the present study's findings suggest that major dietary patterns are associated with different metabolic phenotypes in middle-aged and elderly Japanese adults. These findings provide useful evidence for maintaining metabolic health through diet regardless of obesity status.

## Data Availability Statement

The raw data supporting the conclusions of this article will be made available by the authors, without undue reservation.

## Ethics Statement

The studies involving human participants were reviewed and approved by Ethical Review Committee of Waseda University. The patients/participants provided their written informed consent to participate in this study.

## Author Contributions

KT, TI, and MH designed the study. KT drafted the manuscript. KT and RK performed the statistical analyses. KT, TI, RK, CU, TK, KS, and KI conducted the investigation. SS, IM, KO, and MH conceived and supervised the study. All authors reviewed and approved the final manuscript.

## Funding

This study was supported in part by grants from the Japan Society for Promotion of Science (JSPS) KAKENHI Scientific Research (B) (MH, Grant Number 18H03198; IM, Grant Number 19H04008; KT, Grant Number 19H04065), and MEXT-Supported Program for the Strategic Research Foundation at Private Universities, 2015–2019 from the Ministry of Education, Culture, Sports, Science, and Technology (Grant Number S1511017).

## Conflict of Interest

The authors declare that the research was conducted in the absence of any commercial or financial relationships that could be construed as a potential conflict of interest.

## Publisher's Note

All claims expressed in this article are solely those of the authors and do not necessarily represent those of their affiliated organizations, or those of the publisher, the editors and the reviewers. Any product that may be evaluated in this article, or claim that may be made by its manufacturer, is not guaranteed or endorsed by the publisher.

## References

[1] Lavie CJ De Schutter A Parto P Jahangir E Kokkinos P Ortega FB. Obesity and prevalence of cardiovascular diseases and prognosis-the obesity paradox updated. Prog Cardiovasc Dis. (2016) 58:537–47. 10.1016/j.pcad.2016.01.008 26826295

[2] Phillips CM. Metabolically healthy obesity: definitions, determinants and clinical implications. Rev Endocr Metab Disord. (2013) 14:219–27. 10.1007/s11154-013-9252-x 23928851

[3] Stefan N Schick F Häring HU. Causes, characteristics, and consequences of metabolically unhealthy normal weight in humans. Cell Metab. (2017) 26:292–300. 10.1016/j.cmet.2017.07.008 28768170

[4] Zheng R Zhou D Zhu Y. The long-term prognosis of cardiovascular disease and all-cause mortality for metabolically healthy obesity: a systematic review and meta-analysis. J Epidemiol Community Health. (2016) 70:1024–31. 10.1136/jech-2015-206948 27126492

[5] Caleyachetty R Thomas GN Toulis KA Mohammed N Gokhale KM Balachandran K. Nirantharakumar K. Metabolically healthy obese and incident cardiovascular disease events among 35 million men and women. J Am Coll Cardiol. (2017) 70:1429–37. 10.1016/j.jacc.2017.07.763 28911506

[6] Yang HK Han K Kwon HS Park YM Cho JH Yoon KH. Obesity, metabolic health, and mortality in adults: a nationwide population-based study in Korea. Sci Rep. (2016) 6:1–10. 10.1038/srep30329 27445194 PMC4957204

[7] Hamer M Stamatakis E. Metabolically healthy obesity and risk of all-cause and cardiovascular disease mortality. J Clin Endocrinol Metab. (2012) 97:2482–8. 10.1210/jc.2011-3475 22508708 PMC3387408

[8] Hu FB. Dietary pattern analysis: a new direction in nutritional epidemiology. Curr Opin Lipidol. (2002) 13:3–9. 10.1097/00041433-200202000-00002 11790957

[9] Rodríguez-Monforte M Sánchez E Barrio F Costa B Flores-Mateo G. Metabolic syndrome and dietary patterns: a systematic review and meta-analysis of observational studies. Eur J Nutr. (2017) 56:925–47. 10.1007/s00394-016-1305-y 27605002

[10] Shab-Bidar S Golzarand M Hajimohammadi M Mansouri S. A posteriori dietary patterns and metabolic syndrome in adults: a systematic review and meta-analysis of observational studies. Public Health Nutr. (2018) 21:1681–92. 10.1017/S1368980018000216 29559025 PMC10261419

[11] Murakami K Shinozaki N Fujiwara A Yuan X Hashimoto A Fujihashi H. Systematic review of principal component analysis-derived dietary patterns in Japanese adults: are major dietary patterns reproducible within a country? Adv Nutr. (2019) 10:237–49. 10.1093/advances/nmy079 30785205 PMC6416039

[12] Ito T Tanisawa K Kawakami R Usui C Ishii K. Micronutrient intake adequacy in men and women with a healthy Japanese dietary pattern. Nutrients. (2019) 12:6. 10.3390/nu12010006 31861388 PMC7019305

[13] Ito T Kawakami R Tanisawa K Miyawaki R Ishii K Torii S. Dietary patterns and abdominal obesity in middle-aged and elderly Japanese adults: Waseda Alumni's Sports, Exercise, Daily Activity, Sedentariness and Health Study (WASEDA'S Health Study). Nutrition. (2019) 58:149–55. 10.1016/j.nut.2018.05.029 30396031

[14] Tanisawa K Ito T Kawakami R Usui C Kawamura T Suzuki K. Association between alcohol dietary pattern and prevalence of dyslipidaemia: WASEDA'S Health Study. Br J Nutr. (2021). 10.1017/S0007114521002671 34256880 PMC9201834

[15] Matthews DR Hosker JP Rudenski AS Naylor BA Treacher DF Turner RC. Homeostasis model assessment: insulin resistance and β-cell function from fasting plasma glucose and insulin concentrations in man. Diabetologia. (1985) 28:412–9. 10.1007/BF00280883 3899825

[16] Armstrong T Bull F. Development of the world health organization global physical activity questionnaire (GPAQ). J Public Health (Bangkok). (2006) 14:66–70. 10.1007/s10389-006-0024-x34639787

[17] Science and Technology Agency. Standard Tables of Food Composition in Japan, 2005. 5th rev. ed. Tokyo: National Printing Bureau. (2005).

[18] Kobayashi S Murakami K Sasaki S Okubo H Hirota N Notsu A. Comparison of relative validity of food group intakes estimated by comprehensive and brief-type self-administered diet history questionnaires against 16 d dietary records in Japanese adults. Public Health Nutr. (2011) 14:1200–11. 10.1017/S1368980011000504 21477414

[19] Kobayashi S Honda S Murakami K Sasaki S Okubo H Hirota N. Both comprehensive and brief self-administered diet history questionnaires satisfactorily rank nutrient intakes in Japanese adults. J Epidemiol. (2012) 22:151–9. 10.2188/jea.JE20110075 22343326 PMC3798594

[20] Matsuzawa Y Nakamura T Takahashi M Ryo M Inoue S Ikeda Y. New criteria for “obesity disease” in Japan. Circ J. (2002) 66:987–92. 10.1253/circj.66.987 12419927

[21] Matsuzawa Y. Metabolic syndrome-definition and diagnostic criteria in Japan. J Atheroscler Thromb. (2005) 12:301. 10.5551/jat.12.301 16394611

[22] Ohkubo T Kikuya M Asayama K Imai Y. A proposal for the cutoff point of waist circumference for the diagnosis of metabolic syndrome in the Japanese population. Diabetes Care. (2006) 29:1986–7. 10.2337/dc06-0842 16873823

[23] Matoba Y Inoguchi T Nasu S Suzuki S Yanase T Nawata H. Optimal cut points of waist circumference for the clinical diagnosis of metabolic syndrome in the Japanese population. Diabetes Care. (2008) 31:590–2. 10.2337/dc07-0802 18071008

[24] Nishimura R Nakagami T Tominaga M Yoshiike N Tajima N. Prevalence of metabolic syndrome and optimal waist circumference cut-off values in Japan. Diabetes Res Clin Pract. (2007) 78:77–84. 10.1016/j.diabres.2007.02.015 17467105

[25] Alberti KGMM Zimmet P Shaw J. Metabolic syndrome - a new world-wide definition. A consensus statement from the International Diabetes Federation. Diabet Med. (2006) 23:469–80. 10.1111/j.1464-5491.2006.01858.x 16681555

[26] Smith GI Mittendorfer B Klein S. Metabolically healthy obesity: facts and fantasies. J Clin Invest. (2019) 129:3978–89. 10.1172/JCI129186 31524630 PMC6763224

[27] Slagter SN Corpeleijn E Van Der Klauw MM Sijtsma A Swart-Busscher LG Perenboom CWM. Dietary patterns and physical activity in the metabolically (un)healthy obese: the Dutch Lifelines cohort study. Nutr J. (2018) 17:1–14. 10.1186/s12937-018-0319-0 29433580 PMC5809859

[28] Suliga E Kozieł D Cieśla E Głuszek S. Association between dietary patterns and metabolic syndrome in individuals with normal weight: a cross-sectional study. Nutr J. (2015) 14:55. 10.1186/s12937-015-0045-9 26025375 PMC4455325

[29] Mirzababaei A Sajjadi SF Ghodoosi N Pooyan S Arghavani H Yekaninejad MS. Relations of major dietary patterns and metabolically unhealthy overweight/obesity phenotypes among Iranian women. Diabetes Metab Syndr Clin Res Rev. (2019) 13:322–31. 10.1016/j.dsx.2018.09.012 30641720

[30] Nikniaz L Abbasalizad Farhangi M Tabrizi JS Nikniaz Z. Association of major dietary patterns and different metabolic phenotypes: a population-based study of northwestern Iran. BMC Endocr Disord. (2019) 19:1–7. 10.1186/s12902-019-0455-3 31795992 PMC6889529

[31] Pereira DLM Juvanhol LL Silva DCG Longo GZ. Dietary patterns and metabolic phenotypes in Brazilian adults: a population-based cross-sectional study. Public Health Nutr. (2019) 22:3377–83. 10.1017/S1368980019002593 31571548 PMC10260465

[32] Rodríguez-Monforte M Flores-Mateo G Sánchez E. Dietary patterns and CVD: a systematic review and meta-analysis of observational studies. Br J Nutr. (2013) 114:1341–59. 10.1017/S0007114515003177 26344504

[33] Li F Hou LN Chen W Chen PL Lei CY Wei Q. Associations of dietary patterns with the risk of all-cause, CVD and stroke mortality: a meta-Analysis of prospective cohort studies. Br J Nutr. (2015) 113:16–24. 10.1017/S000711451400289X 25430485

[34] Nanri A Mizoue T Shimazu T Ishihara J Takachi R Noda M. Dietary patterns and all-cause, cancer, and cardiovascular disease mortality in Japanese men and women: the Japan public health center-based prospective study. PLoS ONE. (2017) 12:1–15. 10.1371/journal.pone.0174848 28445513 PMC5405917

[35] Morimoto A Ohno Y Tatsumi Y Mizuno S Watanabe S. Effects of healthy dietary pattern and other lifestyle factors on incidence of diabetes in a rural Japanese population. Asia Pac J Clin Nutr. (2012) 21:601–8. 10.6133/apjcn.2012.21.4.16 23017319

[36] Arisawa K Uemura H Yamaguchi M Nakamoto M Hiyoshi M Sawachika F. Associations of dietary patterns with metabolic syndrome and insulin resistance : a cross-sectional study in a Japanese population. J ofMedical Investig. (2014) 61:333–44. 10.2152/jmi.61.333 25264052

[37] Nanri A Mizoue T Yoshida D Takahashi R Takayanagi R. Dietary patterns and A1C in japanese men and women. Diabetes Care. (2008) 31:1568–73. 10.2337/dc08-0297 18443193 PMC2494650

[38] Filippini T Naska A Kasdagli MI Torres D Lopes C Carvalho C. Potassium intake and blood pressure: a dose-response meta-analysis of randomized controlled trials. J Am Heart Assoc. (2020) 9:e015719. 10.1161/JAHA.119.015719 32500831 PMC7429027

[39] Aburto NJ Hanson S Gutierrez H Hooper L Elliott P Cappuccio FP. Effect of increased potassium intake on cardiovascular risk factors and disease: systematic review and meta-analyses. BMJ. (2013) 346:1–19. 10.1136/bmj.f1378 23558164 PMC4816263

[40] Reynolds A Mann J Cummings J Winter N Mete E Te Morenga L. Carbohydrate quality and human health: a series of systematic reviews and meta-analyses. Lancet. (2019) 393:434–45. 10.1016/S0140-6736(18)31809-9 30638909

[41] Eslick GD Howe PRC Smith C Priest R Bensoussan A. Benefits of fish oil supplementation in hyperlipidemia: a systematic review and meta-analysis. Int J Cardiol. (2009) 136:4–16. 10.1016/j.ijcard.2008.03.092 18774613

[42] Leslie MA Cohen DJA Liddle DM Robinson LE Ma DWL. A review of the effect of omega-3 polyunsaturated fatty acids on blood triacylglycerol levels in normolipidemic and borderline hyperlipidemic individuals. Lipids Health Dis. (2015) 14:53. 10.1186/s12944-015-0049-7 26048287 PMC4488064

[43] Sacks FM Svetkey LP Vollmer WM Appel LJ Bray GA Harsha D. Effects on blood pressure of reduced Dietary Sodium and the Dietary Approaches to Stop Hypertension (DASH) diet. N Engl J Med. (2001) 344:3–10. 10.1056/nejm200101043440101 11136953

[44] Trichopoulou A Lagiou P. Healthy traditional Mediterranean diet: an expression of culture, history, and lifestyle. Nutr Rev. (1997) 55:383–9. 10.1111/j.1753-4887.1997.tb01578.x 9420448

[45] Micha R Khatibzadeh S Shi P Fahimi S Lim S Andrews KG. Global, regional, and national consumption levels of dietary fats and oils in 1990 and 2010: a systematic analysis including 266 country-specific nutrition surveys. BMJ. (2014) 348:1–20. 10.1136/bmj.g2272 24736206 PMC3987052

[46] Song Y Joung H. A traditional Korean dietary pattern and metabolic syndrome abnormalities. Nutr Metab Cardiovasc Dis. (2012) 22:456–62. 10.1016/j.numecd.2010.09.002 21215606

[47] Roerecke M Kaczorowski J Tobe SW Gmel G Hasan OSM Rehm J. The effect of a reduction in alcohol consumption on blood pressure: a systematic review and meta-analysis. Lancet Public Heal. (2017) 2:e108–20. 10.1016/S2468-2667(17)30003-8 29253389 PMC6118407

[48] Van De Wiel A. The effect of alcohol on postprandial and fasting triglycerides. Int J Vasc Med. (2012) 2012:862504. 10.1155/2012/862504 21961068 PMC3179875

[49] Brien SE Ronksley PE Turner BJ Mukamal KJ Ghali WA. Effect of alcohol consumption on biological markers associated with risk of coronary heart disease: systematic review and meta-analysis of interventional studies. BMJ. (2011) 342:480. 10.1136/bmj.d636 21343206 PMC3043110

[50] Jean-Pierre Despres Lemieux I. Abdominal obesity and metabolic syndrome. Nature. (2006) 444:881–887. 10.1038/nature05488 17167477

[51] Yamagishi K Iso H. The criteria for metabolic syndrome and the national health screening and education system in Japan. Epidemiol Health. (2017) 39:e2017003. 10.4178/epih.e2017003 28092931 PMC5343105

[52] Knopp RH Zhu X Bonet B. Effects of estrogens on lipoprotein metabolism and cardiovascular disease in women. Atherosclerosis. (1994) 110:S83–91. 10.1016/0021-9150(94)05379-W 7857390

[53] De Aloysio D Gambacciani M Meschia M Pansini F Modena AB Bolis PF. The effect of menopause on blood lipid and lipoprotein levels. Atherosclerosis. (1999) 147:147–53. 10.1016/S0021-9150(99)00315-9 10525136

